# Importance of adhesins in virulence of *Paracoccidioides* spp.

**DOI:** 10.3389/fmicb.2015.00303

**Published:** 2015-04-10

**Authors:** Haroldo C. de Oliveira, Julhiany de Fátima da Silva, Liliana Scorzoni, Caroline M. Marcos, Suelen A. Rossi, Ana C. A. de Paula e Silva, Patrícia A. Assato, Rosângela A. M. da Silva, Ana M. Fusco-Almeida, Maria J. S. Mendes-Giannini

**Affiliations:** Laboratório de Micologia Clínica, Departamento de Análises Clínicas, Faculdade de Ciências Farmacêuticas, UNESP – Universidade Estadual PaulistaAraraquara, Brazil

**Keywords:** *Paracoccidioides* spp., virulence, adhesion, adhesins

## Abstract

Members of the *Paracoccidioides* genus are the etiologic agents of paracoccidioidomycosis (PCM). This genus is composed of two species: *Paracoccidioides brasiliensis* and *Paracoccidioides lutzii*. The correct molecular taxonomic classification of these fungi has created new opportunities for studying and understanding their relationships with their hosts. *Paracoccidioides* spp. have features that permit their growth under adverse conditions, enable them to adhere to and invade host tissues and may contribute to disease development. Cell wall proteins called adhesins facilitate adhesion and are capable of mediating fungi-host interactions during infection. This study aimed to evaluate the adhesion profile of two species of the genus *Paracoccidioides*, to analyze the expression of adhesin-encoding genes by real-time PCR and to relate these results to the virulence of the species, as assessed using a survival curve in mice and in *Galleria mellonella* after blocking the adhesins. A high level of heterogeneity was observed in adhesion and adhesin expression, showing that the 14-3-3 and enolase molecules are the most highly expressed adhesins during pathogen-host interaction. Additionally, a survival curve revealed a correlation between the adhesion rate and survival, with *P. brasiliensis* showing higher adhesion and adhesin expression levels and greater virulence when compared with *P. lutzii*. After blocking 14-3-3 and enolase adhesins, we observed modifications in the virulence of these two species, revealing the importance of these molecules during the pathogenesis of members of the *Paracoccidioides* genus. These results revealed new insights into the host-pathogen interaction of this genus and may enhance our understanding of different isolates that could be useful for the treatment of this mycosis.

## Introduction

Members of *Paracoccidioides* spp. are dimorphic fungi and the etiological agents of paracoccidioidomycosis (PCM), a systemic mycosis affecting people in Latin America. Brazil has the largest number of endemic PCM areas in the world (Franco et al., 1993).

Matute et al. (2006) classified the *Paracoccidioides* genus into three phylogenetic species: S1, PS2, and PS3. These species have different geographic distributions: S1 is a paraphyletic group found in Brazil, Argentina, Paraguay, Peru, and Venezuela; PS2 is a monophyletic group found in Brazil and Venezuela; and PS3 is a monophyletic group found only in Colombia. Carrero et al. (2008) classified these isolates into two phylogenetic species, S1 and PS3, which were previously described by Matute et al. (2006), and also classified isolate Pb01 as a new phylogenetic species. Teixeira et al. (2009a) proposed that the Pb01 isolate was a new species, *P. lutzii*. The newly revised molecular taxonomy for these fungi has created new opportunities for studying and understanding their eco-epidemiological relationships with their hosts (Bagagli et al., 2006; Teixeira et al., 2009a). *P. lutzii* and *P. brasiliensis* are found in the west-central and in the southern/southeastern regions of Brazil, respectively (Gegembauer et al., 2014).

The agents of systemic mycoses have some properties that permit their growth in the adverse conditions of the host and may contribute to the development of disease (Casadevall and Pirofski, 1999). *Paracoccidioides* spp. have mechanisms that enable them to adhere to and invade barriers imposed by the host tissues (Mendes-Giannini et al., 1994, 2006). The fungi synthesize several substances that participate directly or indirectly in the parasite-host relationship (Mendes-Giannini et al., 2000). Therefore, the successful colonization of the host tissues by the fungus is a complex event, usually involving a pathogen ligand and a host cell receptor. The microorganism has three host components with which it can interact: products secreted by the cell, surfaces of the host cell, and proteins of the extracellular matrix (ECM), such as type I and IV collagens, fibronectin and laminin (Mendes-Giannini et al., 2006). The understanding and identification of molecules involved in the adhesion of microorganisms to different substrates in the host can aid in the discovery of efficient treatments for systemic mycoses.

*Paracoccidioides* virulence is a multifaceted event involving the expression of multiple genes at different stages of infection (da Silva et al., 2013). Several adhesins have been described in *Paracoccidioides* spp. and these characterizations have most often been performed using the Pb18 and Pb01 strains. The importance of the following adhesins in fungus-host interactions has been examined: GP43 (Hanna et al., 2000; Mendes-Giannini et al., 2006), 14-3-3 protein (30 kDa) (Andreotti et al., 2005), glyceraldehyde-3-phosphate dehydrogenase (GAPDH) (Barbosa et al., 2006), triosephosphate isomerase (TPI) (Pereira et al., 2007), enolase (Donofrio et al., 2009; Nogueira et al., 2010; Marcos et al., 2012), and malate synthase (da Silva Neto et al., 2009) using different methodologies like ligand affinity binding, inhibition using antibodies, biding, biding competition, Far-Western blot, ELISA, and Western blot assays.

Besides its direct participation in adhesion, most of the described adhesins for *Paracoccidoides* spp. are enzymes that participate in the glycolytic pathway, tricarboxylic acid cycle and glyoxylate cycle in Paracoccidioides spp. acting as “moonlighting proteins” (Marcos et al., 2014). Recent studies have shown the presence of these enzymes in extracellular vesicles produced by the fungi as well as being present in the cell wall, wherein blocking them leads to a decrease in the ability of the fungi to adhere to cellular host components (Puccia et al., 2011; Vallejo et al., 2011, 2012a,b).

The presence of these enzymes at the yeast cell surface in the absence of a conventional N-terminal signal sequence responsible for targeting the protein into the classical secretory pathway is an intriguing question in the study of the paracoccidioimycosis. Some of these enzymes could behave as anchorless adhesins, which bind to the cell wall and allows direct interaction with the host (da Silva Neto et al., 2009). However, its known that in the N-terminal half of the *C. albicans* GAPDH polypeptide encoded by the TDH3 gene, for example, is able to direct its incorporation into the yeast cell wall (Delgado et al., 2003) but, in the case of the fungi from the *Paracoccidioides* genus, more studies are needed to identify putative signals related to its cell wall targeting.

How they interact with the host cells is another intriguing question. Marcos et al. (2012) demonstrated that the *P. brasiliensis* enolase has a plasminogen-binding motif (RGD) (_254_FYKADEKKY_262_), that is involved in the direct interaction with the host cell surface. It was shown, that when the RGD motif was added as a competitor in the binding assay of enolase to pneumocytes, the binding of the enzyme to the host cells decreased 10%, demonstrating the role of this motif in the enzyme binding to cellular components of the host, however more studies should be done to better understand how these adhesins interacts with the host.

The virulence of fungi may be evaluated using murine models. More recently, the larvae of *G. mellonella* are increasingly being used as an infection model for studying the virulence factors and pathogenesis of many fungal and bacterial pathogens (Cook and McArthur, 2013). Murine models are still considered the gold standard for the study of pathogenesis; however, economic, logistical and ethical considerations limit the use of mammalian host models of infection, especially when the experiment requires the analysis of a large number of strains (Jacobsen, 2014). Thomaz et al. (2013) used *G. mellonella* to evaluate the efficacy of this model for the study of the dimorphic fungi *P. lutzii* and *Histoplasma capsulatum* and concluded that this is a potentially useful model for studying the virulence of dimorphic fungi.

Thus, this study aims to investigate adhesins in the two species of the *Paracoccidioides* genus and to understand how the capacity of these species to express adhesins affects their virulence.

## Materials and methods

### Ethics statements

Animal experiments were performed according to Brazilian Federal Law 11.794, which has established procedures for the use of animals in scientific research, and to state law, which has established the code of animal protection of the State of São Paulo. For this study, male BALB/C and C57BL/6 mice (3–4 weeks old) were used, and all efforts were made to minimize the suffering of the animals. The experiments were approved by the Ethics Committee on Animal Experiments of the Faculty of Pharmaceutical Sciences of Araraquara—UNESP FCFAr (Case 10/2011/CEUA/FCF).

### Microorganisms

Experiments were performed with two isolates belonging to two species of the genus *Paracoccidioides*: *P. brasiliensis* (Pb18) and *P. lutzii* (Pb01). Pb18 is representative of the major phylogenetic group S1 and has been extensively used in the literature due to its demonstrated virulence in mice when inoculated by the intraperitoneal, intratracheal and intravenous routes (Calich et al., 1998). Pb01 is a clinical isolate from an acute form of PCM in an adult male and is the most thoroughly studied isolate at the molecular level, as shown by an extensive analysis of its transcriptome that yielded ESTs covering approximately 80% of the estimated genome of *P. lutzii* (Felipe et al., 2005b). These isolates were maintained in the mycelial phase (M) and were transferred to Fava-Netto medium supplemented with fetal bovine serum to revert to the yeast phase (L) before the experiments. After reversion, isolates were maintained in Fava-Netto medium without any supplements. Consecutive subcultures were generated each week. The isolates were used for experiments after the 4th subculture. Before the experiments, the fungi were growth for 4 days in brain heart infusion medium (BHI) supplemented with glucose (2%) at 37°C with shaking at 250 rpm.

### Adhesion to A549 pneumocytes of *P. brasiliensis* and *P. lutzii*

Cells from the A549 epithelial lineage (type II pneumocytes from the Rio de Janeiro Cell Bank) were grown in 6-well cell plates in HAM F-12 medium supplemented with 10% fetal bovine serum. After monolayer formation, the cells were washed three times with sterile phosphate-buffered saline (PBS) and inoculated with a *Paracoccidioides* spp. inoculum of 5 × 10^6^ cells/mL. The standard inoculum of *Paracoccidioides* spp. in PBS had previously been incubated for 15 min at 37°C with 10 μM CFSE [5(6)-carboxyfluorescein diacetate N-succinimidyl ester], which stains the fungal cell wall, washed and resuspended in PBS. The infected cells were then incubated for 2–5 h at 36.5°C and 5% CO_2._After incubation, the cells were removed from the plates using 0.2% ATV-trypsin solution and 0.02% Versene (Adolfo Lutz). The trypsinized cells were washed in medium containing fetal bovine serum, centrifuged and fixed with 4% paraformaldehyde. The fixed cells were then evaluated using flow cytometry (FACSCanto, BD) to determine the amount of adherent fungi and to thereby determine the infective capacity of the different species of the *Paracoccidioides* genus. Experiments were performed in triplicate with three independent experiments for each species. Statistical analyses were performed using One-Way ANOVA with Tukey's coefficient. The results of the statistical analyses were considered significant when the *p*-value was <0.05. Analyses and the construction of graphs were performed using Prism 5 software (GraphPad Software Inc.).

### Adhesion of *P. brasiliensis* and *P. lutzii* to the extracellular matrix components laminin, fibronectin and type I and type IV collagen

Twenty-four-well plates (Corning®) were coated with the ECM components laminin, fibronectin and type I and IV collagen (Sigma Aldrich) at 50 μg/ml for 18 h at 4°C and 1 h at room temperature. After the sensitization period, the plates were washed three times with PBS. For the adhesion assay, according to Oliveira et al. (2014), inocula of different phylogenetic species of *Paracoccidioides* (5 × 10^6^ cells/mL) were prepared in PBS. Then, 500 μL of these inocula were added to the plate, which had already been coated with components of the ECM, as described above. The plates were incubated at 37°C for 2 h. After the incubation, the supernatant was removed, and the plates were washed three times with PBS. To remove the fungus that adhered to the different ECM components, 300 μL of 0.2% ATV-trypsin solution and 0.02% Versene (Adolfo Lutz) was added to each well. Trypsin suspensions were collected and centrifuged at 2500 rpm at 4°C. Subsequently, the supernatants were discarded, and 500 μL of FACSFlow was added for subsequent cell counting by flow cytometry (FACSCanto, BD) using the BD FACSDiva software (BD). These experiments were also performed in triplicate, with three independent experiments for each species. Statistical analyses were performed using one-way ANOVA with Tukey's coefficient. The results of the statistical analyses were considered significant when the *p*-value was <0.05. Analyses and the construction of graphs were performed using Prism 5 software (GraphPad Software Inc.).

### Expression analysis of the genes encoding adhesins by real-time PCR

The expression of genes encoding seven adhesins, which were previously described in *Paracoccidioides* spp., was evaluated in the different species. Specific primers for each gene were synthesized (Table 1). The L34 gene was used as the housekeeping gene in the analysis.

**Table 1:** **Primers used for the real-time PCR assays**.

**Gene**	**Primers**	**Product length (bp)**
Enolase	Sense	130
	5′-TAGGCACCCTCACTGAATCC-3′	
	Antisense	
	5′-GCTCTCAATCCCACAACGAT-3′	
GADPH	Sense	129
	5′-AAATGCTGTTGAGCACGATG-3′	
	Antisense	
	5′-CTGTGCTGGATATCGCCTTT-3′	
GP43	Sense	135
	5′-CTTGTCTGGGCCAAAAACTC-3′	
	Antisense	
	5′-CGGGACTGGGAGTGAGATAT-3′	
Malate synthase	Sense	101
	5′-GTTCCCTTCATGGATGCCTA-3′	
	Antisense	
	5′-TCTTTGATGGGGATTTGAGC-3′	
Triosephosphate isomerase	Sense	118
	5′-CCTTACGGCAGAATGACGTT-3′	
	Antisense	
	5′-GCCATTTCCATGTCAGGTCT-3′	
14-3-3	Sense	76
	5′ -GTTCGCTCTTGGAGACAAGC-3′	
	Antisense	
	5′ -AGCAACCTCAGTTGCGTTCT-3′	
L34 (endogenous gene)	Sense	149
	5′-TGTCTACACTGCGCAAGGAC-3′	
	Antisense	
	5′-ATGTGTTGGTGGGAGAGGAG-3′	

For this experiment, groups of 10 male BALB/c mice were infected by tail vein injection with the following *Paracoccidioides* isolates: Pb18 (*P. brasiliensis*) and Pb01 (*P. lutzii)*. The blood of the animals was collected for RNA extraction (Bailão et al., 2006) after 10 min and 1 h of infection, and gene expression was investigated. In this study, we analyzed the expression of adhesins at earlier time points (10 min and 1 h) than those evaluated for adhesion studies (2 and 5 h) to determine the expression of adhesins in response to the first contact of the fungus with the host (infected BALB/C mice) because adhesins are molecules of great importance during the initial contact of the fungus with the host.

RNA from the fungi recovered from mouse blood was extracted using the TRIzol method (Invitrogen Life Technologies, Carlsbad, CA). First-strand cDNA synthesis was performed using reverse transcriptase (RevertAid™ H Minus Reverse Transcriptase, Fermentas, Canada) and 1 μg of total RNA.

The reaction mixtures contained 1 μL of cDNA, 12.5 μL of Maxima® SYBR Green/ROX qPCR Master Mix (2X) (Fermentas, Canada), and 0.5 μM of each primer, and the volume was brought to 25 μL with nuclease-free water. The reaction program was 50°C for 2 min, 95°C for 10 min, and 40 cycles of 95°C for 15 s followed by annealing and synthesis at 60°C for 1 min. Following the PCR, a melting curve analysis was performed, which confirmed that the signal corresponded to a single PCR product. Reactions were performed in an Applied Biosystems 7500 cycler. The data were analyzed using the 2^−ΔΔCT^ method. The cycle threshold values for the triplicate PCRs of each RNA sample were averaged, and then the 2^−ΔΔCT^ values were calculated using the control gene 60S ribosomal L34, which is a constitutive housekeeping gene that is widely used as a control in different studies of paracoccidioidomycosis (Felipe et al., 2005a; Bailão et al., 2006, 2007; Brito et al., 2011; Parente et al., 2011; Peres da Silva et al., 2011; Lima et al., 2014). A sample that contained all reagents except *P. brasiliensis* cDNA was used as a negative control. After 40 rounds of amplification, no PCR products were detected in this reaction. Two biological samples were obtained for each studied situation and for each sample, and three independent experiments were performed. As a control experiment, an RNA sample was obtained from the blood of mice not infected with *P. brasiliensis*. Before beginning the experiments, all of the primers were evaluated to assess the efficiency of the amplifications by analyzing the standard curves using serial dilutions of cDNA as a template and determining the slope value. Subsequently, we used this value to evaluate the efficiency using the formula Efficiency = 10^(−1/slope^)-1. After the experiments, the amplification products (in 1.5% agarose gels) and the dissociation curves were analyzed to ensure the amplification of only one PCR product. Statistical analyses were performed using One-Way ANOVA with Tukey's coefficient. The results of the statistical analyses were considered significant when the *p*-value was <0.05. Analyses and the construction of graphs were performed using Prism 5 software (GraphPad Software Inc.).

### Analysis of the relationship between adhesion and virulence in different species of the genus *Paracoccidioides*

For this analysis, aliquots (5 × 10^6^ cells/ml) of each isolate of the genus were intratracheally inoculated into C57BL/6 male mice. The mice were anesthetized by administration of a combination of 250 μL of 10 mg/kg xylazine and 80 mg/kg ketamine hydrochloride. A small incision was made in the trachea region, and 0.05 mL of fungal suspension inoculum was injected using a 1.0 mL syringe. The incision was sutured, and the animals were kept warm to avoid hypothermia due to the action of the anesthetic. After inoculation, the mice were observed for mortality until 200 days post-infection. Each group contained eight mice, and a control group was inoculated with only PBS. For the survival curves, the statistical analyses were performed using the Mantel-Cox test in GraphPad Prism software, and the *p*-value was set at *p* < 0.05.

### Evaluation of the role of the two major expressed adhesins, 14-3-3 and enolase, in the adhesion of the *Paracoccidioides* genus to A549 pneumocytes

This experiment was made in the same way as described in the section Adhesion to A549 pneumocytes of *P. brasiliensis* and *P. lutzii* with a modification. After the treatment of the *Paracoccidioides* spp. cells with 10 uM CFSE, the cells were washed, resuspended in PBS and treated with 14-3-3 and enolase rabbit antisera (1:100) for 1 h at 37°C. After this new treatment, the cells were washed, resuspended in PBS and added to A549 pneumocytes culture for 2 and 5 h. These experiments were also performed in triplicate, with three independent experiments for each species. Statistical analyses were performed using One-Way ANOVA with Tukey's coefficient. As controls, we used *Paracoccidioides* spp. without any treatment and treated with rabbit antisera (1:100) for 1 h at 37°C. The results of the statistical analyses were considered significant when the *p*-value was <0.05. Analyses and the construction of graphs were performed using Prism 5 software (GraphPad Software Inc.).

### Evaluation of the role of the two major expressed adhesins, 14-3-3 and enolase, in the virulence of the *Paracoccidioides* genus using *G. mellonella*

For this analysis, *P. brasiliensis* (Pb18) and *P. lutzii* (Pb01) were treated with 14-3-3 and enolase rabbit antisera (1:100) for 1 h at 37°C. The 14-3-3 and enolase rabbit antisera used in this study were obtained and used in three previous studies performed by our group (Donofrio et al., 2009; Marcos et al., 2012; da Silva et al., 2013). After treatment, the inocula were washed 3 times with PBS and inoculated into the larvae of *G. mellonella*. The rearing of *G. mellonella* was performed according to Ramarao et al. (2012). The larvae were fed wax and pollen and maintained at 25°C until they reached 100–200 mg in weight. Larvae without color alterations and with the appropriate weight were selected in groups of 16 larvae, placed in Petri dishes and incubated at 37°C in the dark on the night before the experiments. The larvae were inoculated through the last left pro-leg using a 10 μL Hamilton syringe (Hamilton, USA) after cleaning the pro-leg with 70% ethanol.

A total of 5 × 10^6^ cells were injected into each larva. Larval death was recorded over the next 7 days and was based on lack of movement after manipulation with forceps. For this experiment, 6 different controls were used: (1) PBS, (2) larvae treated with 14-3-3 rabbit antisera alone (not infected with fungi), (3) larvae treated with enolase rabbit antisera alone (not infected with fungi), (4) larvae treated with rabbit antisera alone (no infection), (5) larvae infected with *P. brasiliensis* and treated with rabbit antisera for 1 h at 37°C, (6) larvae infected with *P. lutzii* and treated with rabbit antisera for 1 h at 37°C, (7) larvae treated with *P. brasiliensis* alone and (8) larvae treated with *P. lutzii*.

For the survival curves, three independent experiments were performed. The statistical analyses were performed using the Mantel-Cox test in GraphPad Prism software, and the *p*-value was set at *p* < 0.05.

The influence of antisera on the survival and morphology of the fungi was evaluated to analyze how these factors interfere with the results found in the adhesion inhibition assays and survival curves. For this, *Paracoccidioides* spp. Pb18 and Pb01 were grown for 7 days in BHI with 2% glucose and with or without 14-3-3, enolase or rabbit antisera at a titer of 1:100. Every 24 h, aliquots of the fungi were obtained, and the morphology and viability were analyzed. To analyze morphology, the fungi were fixed with 1% paraformaldehyde, stained with calcofluor white stain (40 μL/mL) (Sigma-Aldrich) and observed using fluorescence microscopy. The viability of the cells was assessed by measuring metabolic activity using a 2,3-bis (2-methoxy-4-nitro-5-sulfophenyl)-5-[(phenylamino) carbonyl]-2H-tetrazolium-hydroxide (XTT) (Sigma) reduction assay as described by Meshulam et al. (1995).

## Results

### Adhesion to A549 cells of *Paracoccidioides* spp.

**Figure 1:**
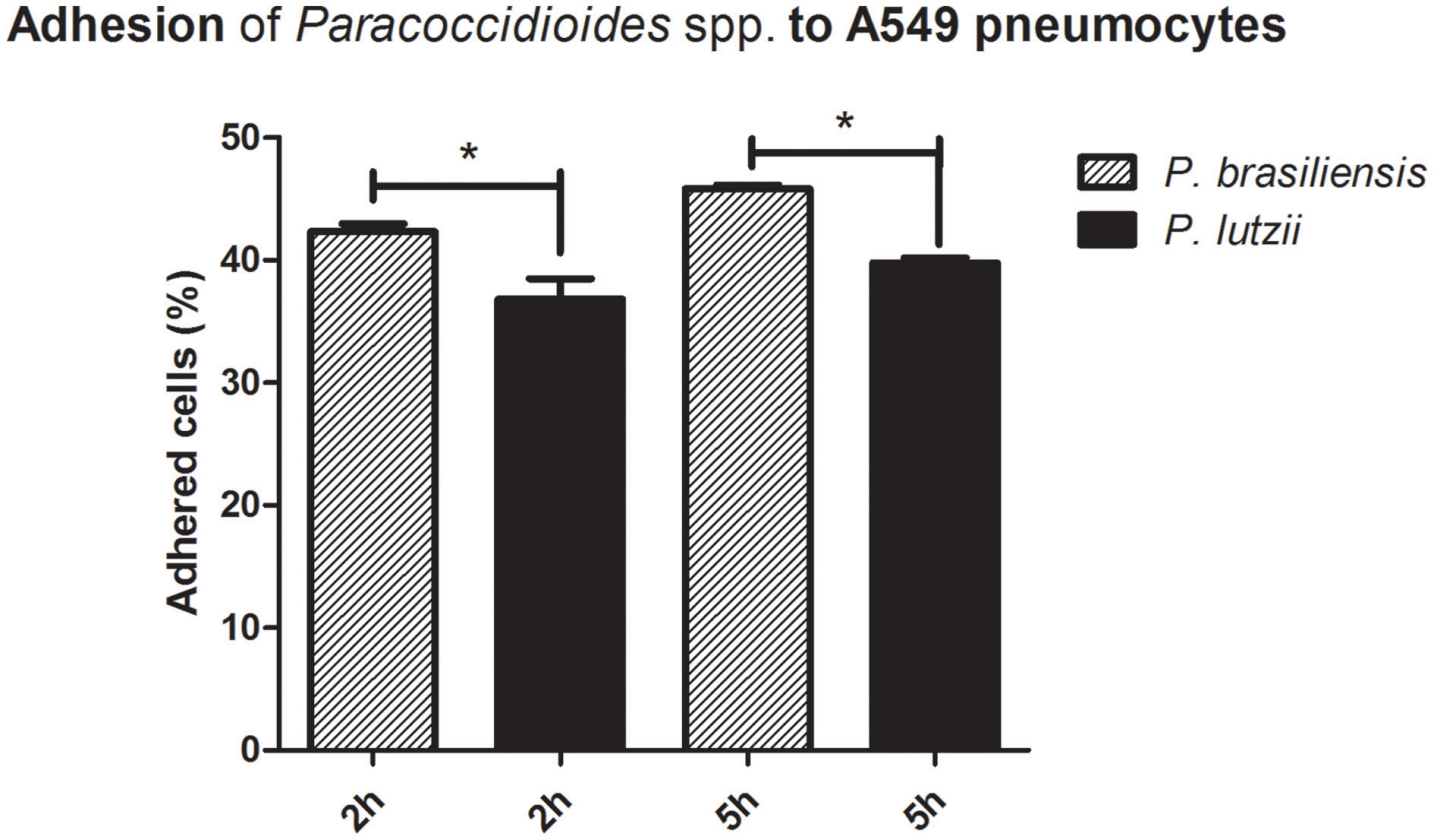
**Adhesion profile to A549 pneumocytes**. Adhesion profiles of *Paracoccidioides brasiliensis* and *Paracoccidioides lutzii* to A549 pneumocytes after 2 and 5 h of infection. ^*^Indicates a statistically significant difference between the adhesion rates of each species, *p* < 0.05.

The results of the adhesion assay with the different species are presented in Figure 1. The adhesion rates were significantly different (*p* < 0.05) when comparing *P. brasiliensis* and *P*. lutzii. After 2 h of interaction, the percentage of adhesion of *P. brasiliensis* was approximately 42.3%, while the adhesion of the *P. lutzii* isolate was approximately 36.8%. After 5 h of infection, the difference in the adhesion rates was still significantly different, with rates of approximately 45.8% in the *P. brasiliensis* isolate and approximately 39.7% in the *P. lutzii* isolate. The adhesion rate may reflect the virulence of each isolate because adhesion is an essential step for successful infection by fungi of the *Paracoccidioides* genus.

### Adhesion of *Paracoccidioides* spp. to the ECM components laminin, fibronectin, and type I and type IV collagen

**Figure 2:**
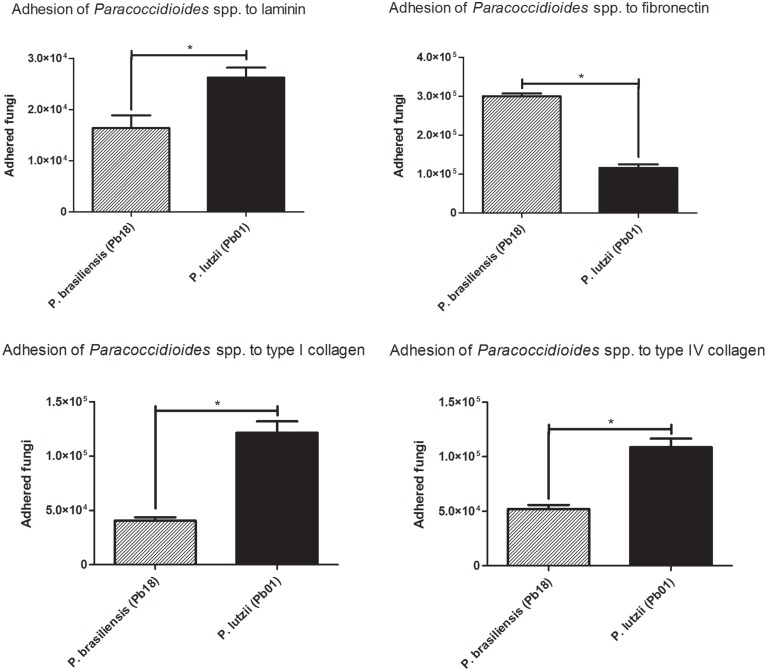
**Adhesion profile to ECM components**. Adhesion profiles of the different species of the genus *Paracoccidioides* to the ECM components (laminin, fibronectin, Type I collagen and Type IV collagen) after 2 h of infection. ^*^Indicates a statistically significant difference in the adherence rate, *p* < 0.05.

These assays were performed with Pb18 (*P. brasiliensis*) and Pb01 (*P. lutzii*) isolates that were placed into contact with ECM components (laminin, fibronectin, and collagens type I and type IV), after which adherent cells were removed and counted by flow cytometry. Three independent experiments were performed in triplicate and statistically evaluated (Figure 2).

The two species appeared to have different affinities for certain extracellular matrix components. Pb18 (*P. brasiliensis*) showed increased adhesion to fibronectin, while Pb01 (*P. lutzii*) was more adherent to type I and IV collagen.

### Expression analysis of genes encoding adhesins by real-time PCR

Before we analyzed the relative expression, the efficiency of the amplifications was evaluated. For this analysis, we had to determine the value of the slope for each tested primer, and we found that the values were close to −3.32 (L34: −3.4; GP43: −3.24; ENO: −3.26; TPI: −3.24; GAPDH: −3.22; 14-3-3: −3.24; MLT: −3.25); thus, the efficiency was greater than 95% (L34: 96%; GP43: 100%; ENO: 100%; TPI: 100%; GAPDH: 100%; 14-3-3: 100%; MLT: 100%). In addition, after the amplification, the amplicons were checked by running them on agarose gels to confirm the size of the expected products. The melting curves presented only one peak, indicating that only one product was amplified (Supplementary Figure 1).

**Figure 3:**
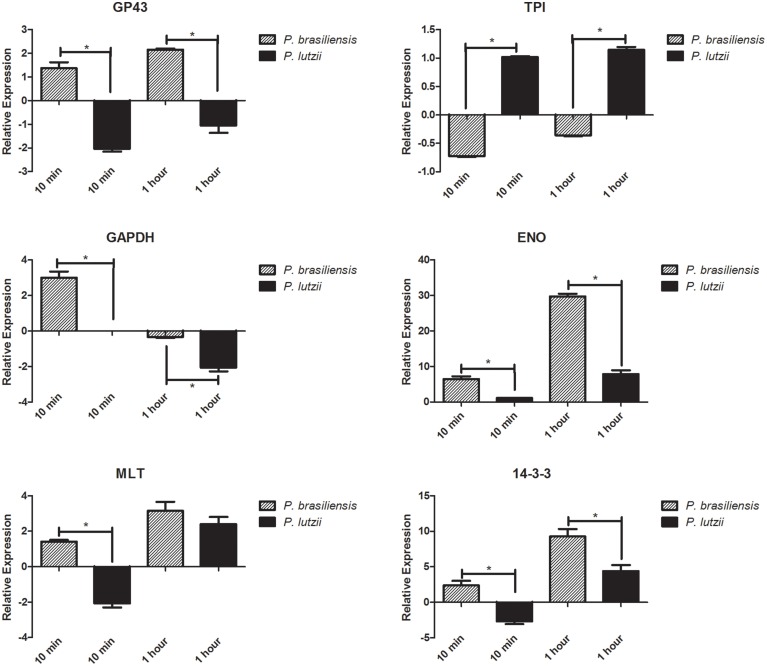
**Relative expression of adhesins**. Relative expression of different genes encoding adhesins in the genus *Paracoccidioides*. ^*^Indicates a statistically significant difference in expression level, *p* < 0.05. The graphs show the normalized value for adhesin gene expression relative to a suitable reference (fungi without contact with the host), which was assigned an arbitrary value of 1.

The relative expression levels of different adhesins in the different *Paracoccidioides* species are presented in Figure 3. Adhesin expression varies, with some adhesins appearing to be more important than others for infection by the different species. Generally, *P. brasiliensis* adhesins are more highly expressed than *P. lutzii* adhesins. Specifically, enolase and 14-3-3 seem to be the most important adhesins for the *Paracoccidioides* genus during its interaction with the host.

During the initial step of the interaction (10 min), only the TPI adhesin was down-regulated in *P. brasiliensis*, while all of the others analyzed were up-regulated during this step. After 1 h of this interaction, the level of expression was still significantly increased in *P. brasiliensis*, showing the importance of these molecules during the interaction of this species with the host. For *P. lutzii*, we observed the opposite, with most of the adhesins down-regulated during the initial step (10 min) of interaction with the host. However, we note that the TPI adhesin was up-regulated and therefore seems to be one of the most important adhesins during the initial process of infection. After 1 h, we were able to observe the same pattern observed in *P. brasiliensis*, namely, a significantly increase in the expression of adhesins.

### Survival curve of C57BL/6 mice for the evaluation of the adhesion/virulence relationship in the genus *Paracoccidioides*

The C57BL/6 mouse strain was chosen because this lineage is more sensitive to *Paracoccidioides* infection and is frequently used for studies examining paracoccidioidomycosis (de Pádua Queiroz et al., 2010; Bernardino et al., 2013; Costa et al., 2013; da Silva et al., 2013).

**Figure 4:**
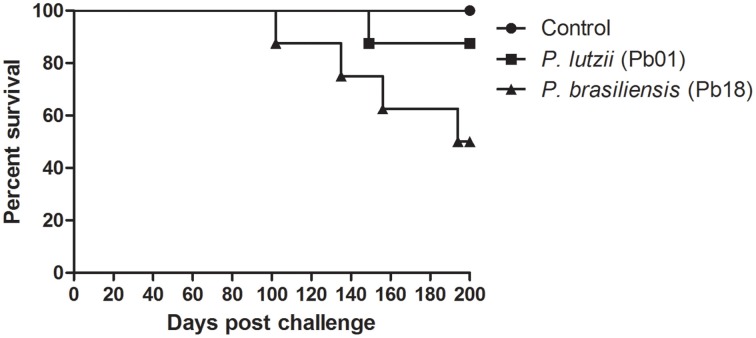
**Virulence of the genus *Paracoccidioides***. Survival curves of C57BL/6 mice infected with *P. brasiliensis* and *P. lutzii* observed for 200 days after infection, showing an increased ability of *P. brasiliensis* to kill mice, with a statistically significant difference relative to the control (*p* < 0.05).

After 200 days of infection, we observed that 4 individuals infected with *P. brasiliensis* died, while only 1 individual infected with *P. lutzii* died, resulting in mouse survival rates of 50 and 90%, respectively (Figure 4).

### Evaluation of the role of the two major expressed adhesins, 14-3-3 and enolase, in the adhesion of the *Paracoccidioides* genus to A549 pneumocytes

**Figure 5:**
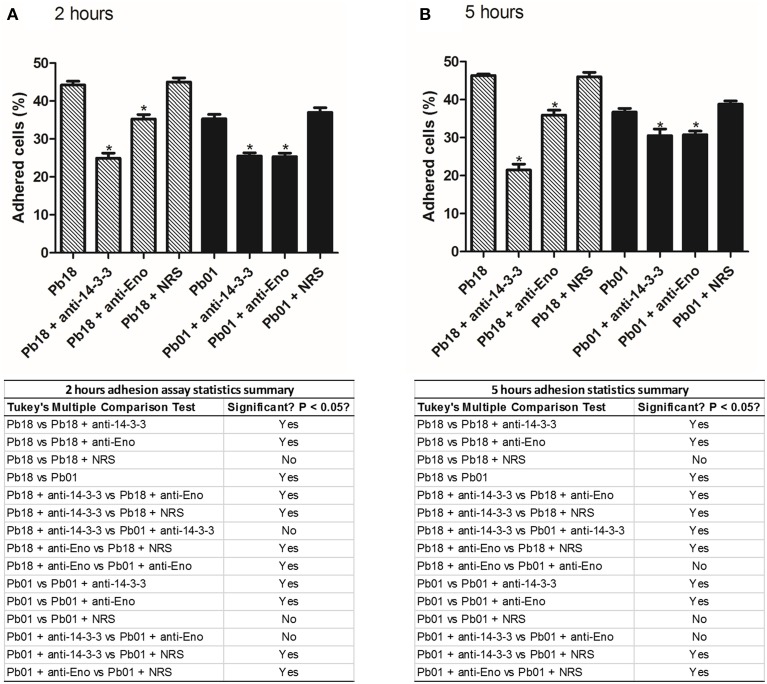
**Influence of adhesins in the adhesion profile of *Paracoccidioides* spp. to pneumocytes A549**. Adhesion profiles of *P. brasiliensis* and *P. lutzii* treated with 14-3-3 and enolase antibodies to A549 pneumocytes after **(A)** 2 h and **(B)** 5 h of infection. As controls, we used *P. brasiliensis* and *P. lutzii* treated with rabbit serum (NRS), and without any treatment. ^*^Indicates statistically significant differences between the adhesion rates, *p* < 0.05. The tables below each graph are summaries of adhesion assay statistics.

It is important to know how these adhesins in fact influence in the adhesion. For this we proposed an experiment where we blocked the two major expressed adhesins 14-3-3 and enolase and, with an adhesion assay, we could measure how much these adhesins influences in the infection process of *Paracoccidioides* spp. After blocking 14-3-3 and enolase we could observe, in 2 and 5 h, that the capacity to adhere to pneumocytes was affected in *Paracoccidioides* spp. (Figure 5). After 2 and 5 h, we could observe that the blocking of 14-3-3 adhesin, lead to a decrease of 19.3 and 24.8 % of *P. brasiliensis* adhesion, respectively. In the case of enolase, adhesion was reduced 8.9 and 10.4% after 2 and 5 h, respectively. For *P. lutzii* we could observe that blocking of 14-3-3 led to 9.8 and 6.2% of the reduction of adhesion after 2 and 5 h. In the case of enolase, adhesion was reduced 9.9 and 5.9% after 2 and 5 h, respectively. These results show that both adhesins are important for full virulence in *Paracoccidioides* spp.

### Evaluation of the role of the two major expressed adhesions, 14-3-3 and enolase, in the virulence of the genus *Paracoccidioides* using *G. mellonella*

**Figure 6:**
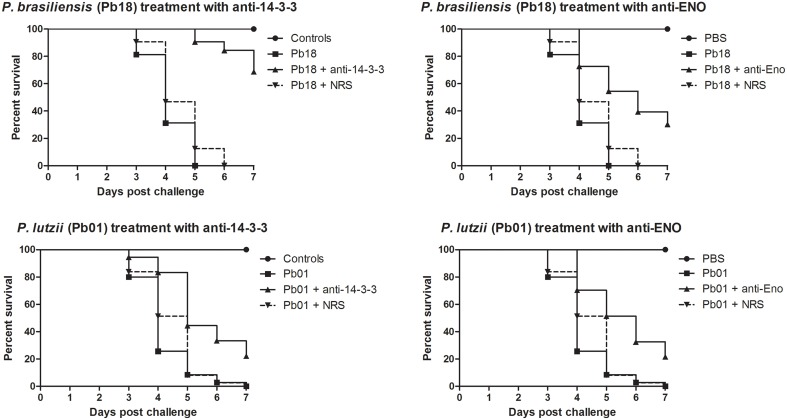
**Influence of adhesins on the virulence of the genus *Paracoccidioides***. Survival curve of *G. mellonella* larvae challenged with *P. brasiliensis* and *P. lutzii* and treated with 14-3-3 and enolase antibodies. Survival was evaluated for 7 days after challenge and showed a reduction in the virulence of the fungi when treated with the antibodies, with a statistically significant difference compared to untreated *P. brasiliensis* and *P. lutzii* (*p* < 0.05). There were 6 different controls: (1) PBS, (2) larvae treated with 14-3-3 antibody alone (not infected with fungi), (3) larvae treated with enolase antibody alone (not infected with fungi), (4) larvae treated with rabbit serum alone (no infection), (5) larvae infected with *P. brasiliensis* and treated with rabbit serum for 1 h at 37°C, and (6) larvae infected with *P. lutzii* and treated with rabbit serum for 1 h at 37°C. All of the controls showed 100% larval survival after the 7-day experiments.

If adhesin expression actually interferes with the virulence of the fungus, blockage of adhesin should affect virulence. To evaluate this hypothesis, we decided to block two of the most highly expressed adhesins in *Paracoccidioides* spp. (based on real-time PCR experiments), 14-3-3 and enolase, using polyclonal antibodies. For this experiment, *P. brasiliensis* and *P. lutzii* were separately treated with 14-3-3 and enolase rabbit antisera (1:100) for 1 h at 37°C. Then, these fungi (5 × 10^6^ cells/larva) were used to infect *G. mellonella* larvae. The virulence of these treated fungi was evaluated for 7 days after fungal challenge. This fungal concentration was previously determined by measuring survival curves with different inoculum sizes for both *P. brasiliensis* and *P. lutzii* (data not shown). Treatment of *P. brasiliensis* and *P. lutzii* with 14-3-3 and enolase polyclonal antibodies led to significant reductions in virulence, as measured by mortality (*p* < 0.05). We observed survival rates of 68.7 and 31.2% for *P. brasiliensis* and 20 and 22% for *P. lutzii* after treatment with 14-3-3 and enolase polyclonal antibodies, respectively (Figure 6). Control larvae showed 100% survival at the end of the 7-day experiment.

These results reflect the influence of these adhesins on the virulence of *Paracoccidioides* spp. because our experimental controls showed us that treating the fungi with the different antisera did not influence survival, with greater than 90% of cells remaining viable for all 7 days of the experiment. Additionally, the morphology of the fungi was not altered during the experiment. All of the results of these control analyses are presented in Supplementary Figure 2. Table 2 shows the standard deviations for each mean value in the graphs.

**Table 2:** **Standard deviations of the *Galleria mellonella* survival curves**.

**(A)**										
**Days**	**Controls**		**Pb 18**		**Pb 18 + Anti-14-3-13**		**Pb 18 +Anti-ENO**		**Pb 18 + NRS**	
	**Mean**	**SEM**	**Mean**	**SEM**	**Mean**	**SEM**	**Mean**	**SEM**	**Mean**	**SEM**
0	100		100		100		100		100	
3	100		81.25	6.90	100		100		90.63	5.15
4	100		31.25	8.19	100		72.73	7.75	46.88	8.82
5	100		0	0	90.6	5.2	54.55	8.67	12.50	5.85
6	100		0	0	84.4	6.4	39.39	8.51	0	0
7	100		0	0	68.8	8.2	30.30	8.00	0	0
**(B)**										
**Days**	**Controls**		**Pb01**		**Pb01 + Anti-14-3-3**		**Pb01 + Anti-ENO**		**Pb01 + NRS**	
	**Mean**	**SEM**	**Mean**	**SEM**	**Mean**	**SEM**	**Mean**	**SEM**	**Mean**	**SEM**
0	100		100		100		100		100	
3	100		80.00	6.76	94.44	3.82	100		83.78	6.06
4	100		25.71	7.39	83.33	6.21	70.27	7.51	51.35	8.22
5	100		8.57	4.73	44.44	8.28	51.35	8.22	8.11	4.49
6	100		2.86	2.82	33.33	7.86	32.43	7.70	2.70	2.67
7	100		0.00	0.00	22.22	6.93	21.62	6.77	0.00	0.00

## Discussion

Studies have demonstrated the ability of *Paracoccidioides* spp. to adhere and invade (Mendes-Giannini et al., 1994), and these characteristics vary depending on the isolate (Hanna et al., 2000). Previous studies have demonstrated that Pb18, now classified as belonging to the S1 phylogenetic species, is the most pathogenic strain in animals (Singer-Vermes et al., 1989) and is more adherent to Vero cells (Hanna et al., 2000). Additionally, after several subcultures, Pb18 loses its adhesion ability, indicating the important relationship between virulence and adhesion. However, upon re-isolation from animals or cell culture, this fungus recovers its ability to adhere to and invade epithelial cells (Andreotti et al., 2005).

In our study, we observed that adhesion was significantly higher for the Pb18 isolate of *P. brasiliensis* than for the Pb01 isolate of *P. lutzii*. The adhesion process reflects the virulence of the isolates because efficient adhesion will culminate in rapid invasion of the host cells, allowing the fungus to escape the host immune system, establish infection, and, in the case of *Paracoccidioides* spp., cause systemic mycosis. After 5 h of infection, the adhesion rate continues to increase, indicating a high capacity to adhere to host cells, as previously observed by our group (Hanna et al., 2000).

These differences in adhesion in different species may reflect differences in the molecules used during interactions with the host. Molecules with greater affinity for different types of ECM components may be associated with the virulence of each isolate and the establishment of the disease in different tissues. Different cell types produce components of the matrix in different quantities, which facilitates the interaction of the fungus with the host depending on the site of the infection. The ability of a microorganism to adhere and invade is recognized as an important factor in pathogenicity. Adhesion implies that the fungus recognizes ligands on the surface of the host cell or a component of the ECM. The adhesion mechanism has been extensively studied in bacteria and pathogenic fungi (de Groot et al., 2013), such as *Candida albicans* (Nobile et al., 2008; Murciano et al., 2012; Puri et al., 2014; Silva et al., 2014; Formosa et al., 2015), *Aspergillus fumigatus* (Upadhyay et al., 2009; Al Abdallah et al., 2012; Xu et al., 2012; Zhao et al., 2012), *Sporothrix schenckii* (Ruiz-Baca et al., 2009; Teixeira et al., 2009b; Sandoval-Bernal et al., 2011), *Coccidioides immitis* (Hung et al., 2002), *Histoplasma capsulatum* (Taylor et al., 2004; Suárez-Alvarez et al., 2010; Pitangui et al., 2012) and *Penicillium marneffei* (Srinoulprasert et al., 2006, 2009; Lau et al., 2013). In a previous study, our group demonstrated that *P. brasiliensis* interacts with human fibronectin (Mendes-Giannini et al., 2006), which confirmed the results of the present study, although there was apparently greater adhesion to laminin. However, these previous assays were performed with extracts, whereas we used whole fungal cells in the present study.

Adhesion is related to the expression of adhesins, and several adhesins have been described in *Paracoccidioides* spp. (Mendes-Giannini et al., 2000, 2008) however, these molecules have not been previously studied to determine their role in the dynamics of infection. In the present study, we analyzed the expression of adhesins at earlier times (10 min and 1 h) than those used for adhesion studies (2 and 5 h) because we aimed to determine whether the fungi respond to host contact and when adhesin expression occurs before the fungi begin the invasion process.

The expression of adhesins in the different species was not constant, and a wide range of expression was observed among the isolates, including a lack of expression. We observed greater expression of certain adhesins, depending on the species and the duration of fungal contact.

The increased expression of GP43 was observed only for *P. brasiliensis* (approximately 1.5-fold higher expression at 10 min and 2.3-fold higher expression at 1 h), while its expression was reduced in *P. lutzii* (approximately 2-fold less expression at 10 min and 1-fold at 1 h). These data are extremely important for the study of paracoccidioidomycosis because the GP43 protein, aside from its role as an adhesin, is the main serological marker for this disease (Puccia and Travassos, 1991). All phylogenetic species cause paracoccidioidomycosis, but a lack of expression of this protein may lead to inaccuracy in serological diagnosis, as demonstrated in some studies (Rocha et al., 2009; Batista et al., 2010; Machado, 2011; Puccia et al., 2011). Our study confirms the data that have already demonstrated the differential role of GP43 in *P. lutzii* specifically that it is not involved in the adhesion of the fungus in this species, and shows that new serological markers for the disease should be screened to avoid making diagnostic mistakes when patients are infected with *P. lutzii* isolates. Additionally, these data confirm that the expression of GP43 is of extreme importance for *P. brasiliensis* virulence, reflecting the importance of this molecule for the diagnosis of patients affected with paracoccidioidomycosis caused by this species.

Another important finding in our study is related to the increase in enolase adhesin expression for the two species, which was approximately 8-fold at 10 min and approximately 35-fold after 1 h of infection with *P. brasiliensis* and was approximately 1-fold after 10 min and approximately 10-fold after 1 h of infection with *P. lutzii*. Recent studies have shown the importance of this protein in *Paracoccidioides* adhesion and virulence. Donofrio et al. (2009) first demonstrated that enolase is a fibronectin adhesin for *P. brasiliensis*, and Nogueira et al. (2010) subsequently described its binding to laminin and collagen type I in addition to fibronectin. Moreover, enolase expressed on the surface of *P. lutzii* acts as a plasminogen receptor, and through this receptor, the fungus can acquire proteolytic activity because of the plasmin generated by this binding event. Plasmin is a key enzyme of the plasminogen system and contributes to the degradation of matrix constituents. This contributes to the pathogenicity of *P. lutzii* because it facilitates tissue invasion by the pathogen. Marcos et al. (2012) demonstrated that fibronectin was the major enolase binder, and it was found at high levels in the fungal cell wall, thereby contributing to *P. brasiliensis* adhesion to the host.

A high level of expression of 14-3-3 adhesin was observed in *P. brasiliensis* after 10 min of interaction, and a significant increase in the expression of 14-3-3 was observed after 1 h for the two species (approximately 12-fold for *P. brasiliensis* and 6-fold for *P. lutzii*). This protein has recently been identified as an important factor in the interaction between *Paracoccidioides* and its host. In a recent study, da Silva et al. (2013) demonstrated that a significant increase in this protein occurs on the pathogen cell wall during its interaction with A549 epithelial cells. This increase suggests an important role for this protein in the fungus-host interaction, leading to a cellular immune response that is important for the success of the fungus in the microenvironment of the host cells. In addition, Vallejo et al. (2011, 2012b) verified that this protein is transported by *P. brasiliensis* vesicles and is secreted into the culture supernatant during *ex vivo* interactions, as described by da Silva et al. (2013).

We observed an increase of triosephosphate isomerase expression in *P. lutzii* of approximately 1-fold after 10 min and 1 h. This protein has only been characterized as an adhesin in isolate Pb01 from *P. lutzii* (Pereira et al., 2007), and it has not been studied in other isolates of the genus *Paracoccidioides*.

Glyceraldehyde 3-phosphate dehydrogenase has also been characterized as an adhesin of *P. lutzii* (Barbosa et al., 2006). In our study, we observed the expression of this adhesin during early interactions between *P. brasiliensis* and the host (an increase of approximately 3.5-fold). For both species, the expression decreased after an hour of interaction (approximately 0.5-fold less expression for *P. brasiliensis* and 2.5-fold less expression for *P. lutzii*), revealing the importance of this protein in early infections with *P. brasiliensis*. Although this finding is controversial, the conditions of our experiment may have influenced these results because this adhesin was described in an *in vitro* experiment, while our study was carried out *in vivo*.

Malate synthase has been described as an adhesin for Pb01 (*P. lutzii*) (da Silva Neto et al., 2009), binding to fibronectin and type I and type IV collagen. Moreover, (de Oliveira et al, 2013) demonstrated that *Paracoccidioides* malate synthase interacts with proteins of different molecular categories, such as those involved in cellular transport, protein biosynthesis, protein degradation and modification and signal transduction, suggesting that this protein plays pleiotropic roles in fungal cells. In the present study, it was expressed less during the early interaction of *P. lutzii* (approximately 2-fold less expression) and of *P. brasiliensis* (expressed approximately 1.5-fold more), but its expression was increased in both species after 1 h of interaction (approximately 4-fold for *P. brasiliensis* and 3-fold for *P. lutzii*).

Apparently, the enolase and 14-3-3 were the most highly expressed adhesins. We observed that the expression of adhesins depends on the duration of the interaction between the host and the fungus. The highest levels of expression for most adhesins were observed in *P. brasiliensis*. This study presents a new perspective on the complex interaction between the *Paracoccidioides* genus and the host and demonstrates the importance of studying different species during the course of infection to understand the molecular arsenal used by the fungus to ensure pathogenic success.

A survival curve was constructed, and *P. brasiliensis* was shown to be more virulent than *P. lutzii*. *P. brasiliensis* was able to kill 50% of the group, while *P. lutzii* killed only 10%. The *Paracoccidioides*-host interaction may cause the induction of apoptosis in the host cells (Mendes-Giannini et al., 2004; Del Vecchio et al., 2009), and this induction can facilitate the establishment and dissemination of the infection in the host. In this way, because *P. brasiliensis* adhere in a more efficient way to pneumocytes than *P. lutzii*, this interaction in an *in vivo* model could lead to the efficient establishment of infection by *P. brasiliensis*, confirming the survival curve observed. These results show that there is an apparent relationship between adherence and virulence in *Paracoccidioides*.

The adhesion of *Paracoccidioides* spp. after blocking 14-3-3 and enolase is significantly affected showing us the importance of these molecules to the infection process of *Paracoccidioides* spp. The 14-3-3 seems to have more influence in this process for *P. brasiliensis* than *P. lutzii*, while enolase, besides seems to have less influence in the adhesion process, the differences found in the adhesions rates are statistically significant. In previously studies, da Silva et al. (2013) made an adhesion inhibition assay of *P. brasiliensis* (Pb18) to pneumocytes A549 and could observe an inhibition in the infection after the treatment of the fungi with the same 14-3-3 antisera, at 2 and 24 h. In a similar way, Donofrio et al. (2009) treated Pb18 cells with enolase antisera and could observe that the adhesion was markedly inhibited by pneumocytes A549 cells after 2 h and 5 h of interaction.

When virulence was evaluated after blocking 14-3-3 and enolase adhesins in *G. mellonella*, we could clearly see that this blocking led to a reduction in the virulence of *P. lutzii* and *P. brasiliensis*, confirming the relationship between adhesins and virulence. After blocking with anti-adhesin antibodies, *P. brasiliensis* virulence was more affected than *P. lutzii* virulence. These data corroborate the real-time PCR results, showing that *P. brasiliensis* expressed higher levels of adhesins than *P. lutzii*, and this elevated expression could be responsible for the higher virulence of *P. brasiliensis* when compared with *P. lutzii*. Besides this, these results also corroborates with the adhesion inhibition assays after blocking 14-3-3 and enolase where we could observe a higher influence of the 14-3-3 in the adhesion of *P. brasiliensis*, however, even with less influence, these two adhesins impact in the virulence of *P. brasiliensis* and *P. lutzii*.

With adhesion blocked, larvae were able to resist the fungal infection and survive. The immune system of *G. mellonella* presents high homology with mammalian organisms and for this reason is considered a suitable model for studying the fungal immune response (Fuchs and Mylonakis, 2006). The cuticle is the first barrier to microorganisms (Champion et al., 2009); moreover, this insect has humoral and cellular defenses. The humoral response is carried out by phenoloxidase, reactive oxygen species and antimicrobial peptides present in the hemolymph, in addition to six different types of cells with phagocytic capacity (Vilmos and Kurucz, 1998; Bergin et al., 2005). Other cellular reactions of the immune system realized by the hemocytes are nodulation and encapsulation; these types of cellular responses occur when the phagocytic cells are unable to phagocytize due to the size of the microorganism, for example (Jiravanichpaisal et al., 2006).

In our results, adherence to A549 pneumocytes by *P. brasiliensis* was more pronounced than in *P. lutzii*. When we examined adhesion to ECM components, we observed that *P. lutzii* was able to adhere more efficiently to three components of the extracellular matrix: laminin, type I collagen and type IV collagen. However, *P. brasiliensis* adhered more efficiently to fibronectin. Fibronectin is an ECM component that is highly important in lung tissue, which is the preferred site of *Paracoccidioides* interaction with the host, because fungal infection occurs through the inhalation of conidia. This makes the lungs the first site of contact between the fungus and the host. Furthermore, *P. brasiliensis* exhibited higher levels of adhesin expression when compared to *P. lutzii*, resulting in greater adherence of these species. These data, when analyzed in relation to the survival curves, make it clear that the greater ability of *P. brasiliensis* to express adhesins leads to greater adhesion and results in increased virulence *in vivo*. Thus, there is a clear relationship between adhesion ability, adhesin expression and fungal virulence in the genus *Paracoccidioides*.

### Conflict of interest statement

The Associate Editor, Helio Takahashi, declares that despite having published with author, Maria Mendes-Giannini, the review process was handled objectively and no conflict of interest exists. The authors declare that the research was conducted in the absence of any commercial or financial relationships that could be construed as a potential conflict of interest.

## Supplementary material

Supplementary Figure S1 **Specific amplification of the studied adhesin genes by real-time PCR. (A)** Melting curve analysis of all of the primers used showing single peaks. **(B)** Agarose gel electrophoresis of the PCR products showing single bands.

Supplementary Figure S2 **Influence of antisera treatment on the survival and morphology of the fungi**. Analysis of the influence of fungi treatment with 14-3-3, enolase and NRS antisera. **(A)** and **(C)** Viability of *P. lutzii* and *P. brasiliensis*, respectively, after treatment with 14-3-3, enolase and NRS antisera. No significant differences were found between the different treatments and the untreated controls. (B) and (D) Analysis of the morphology of *P. lutzii* and *P. brasiliensis*, respectively, after treatment with 14-3-3, enolase and NRS antisera (image magnification: 1000x).
