# Corrigendum: Importance of adhesins in virulence of *Paracoccidioides* spp.

**DOI:** 10.3389/fmicb.2015.00431

**Published:** 2015-05-05

**Authors:** Haroldo Cesar de Oliveira

**Affiliations:** Laboratório de Micologia Clínica, Departamento de Análises Clínicas, Faculdade de Ciências Farmacêuticas, UNESP-Universidade Estadual Paulista Araraquara, Brazil

**Keywords:** virulence, adhesion, adhesins, *Paracoccidioides lutzii*, *Paracoccidioides brasiliensis*

One of the funding sources was omitted from the Acknowledgments list. The corrected list is as follows. This work was supported by the Brazilian organizations Fundação de Apoio à Pesquisa do Estado de São Paulo (FAPESP) 2011/18038-9, Rede Nacional de Métodos Alternativos—Conselho Nacional de Desenvolvimento Científico e Tecnológico (RENAMA-CNPq) 403586/2012-7 and Programa de Apoio ao Desenvolvimento Científico da Faculdade de Ciências Farmacêuticas da UNESP (PADC/FCF). We thank Daniella Sayuri Yamazaki for support with the animal experiment and Dr. Isabel Cristiane da Silva for all the support with A549 pneumocytes culture.

## Conflict of interest statement

The author declares that the research was conducted in the absence of any commercial or financial relationships that could be construed as a potential conflict of interest.

