# Supplementary figures and images for: Importance of adhesins in virulence of Paracoccidioides spp
Source: Front Microbiol. 2015 Apr 10;6:303. doi: 10.3389/fmicb.2015.00303 (PMC4392702; doi:10.3389/fmicb.2015.00303)

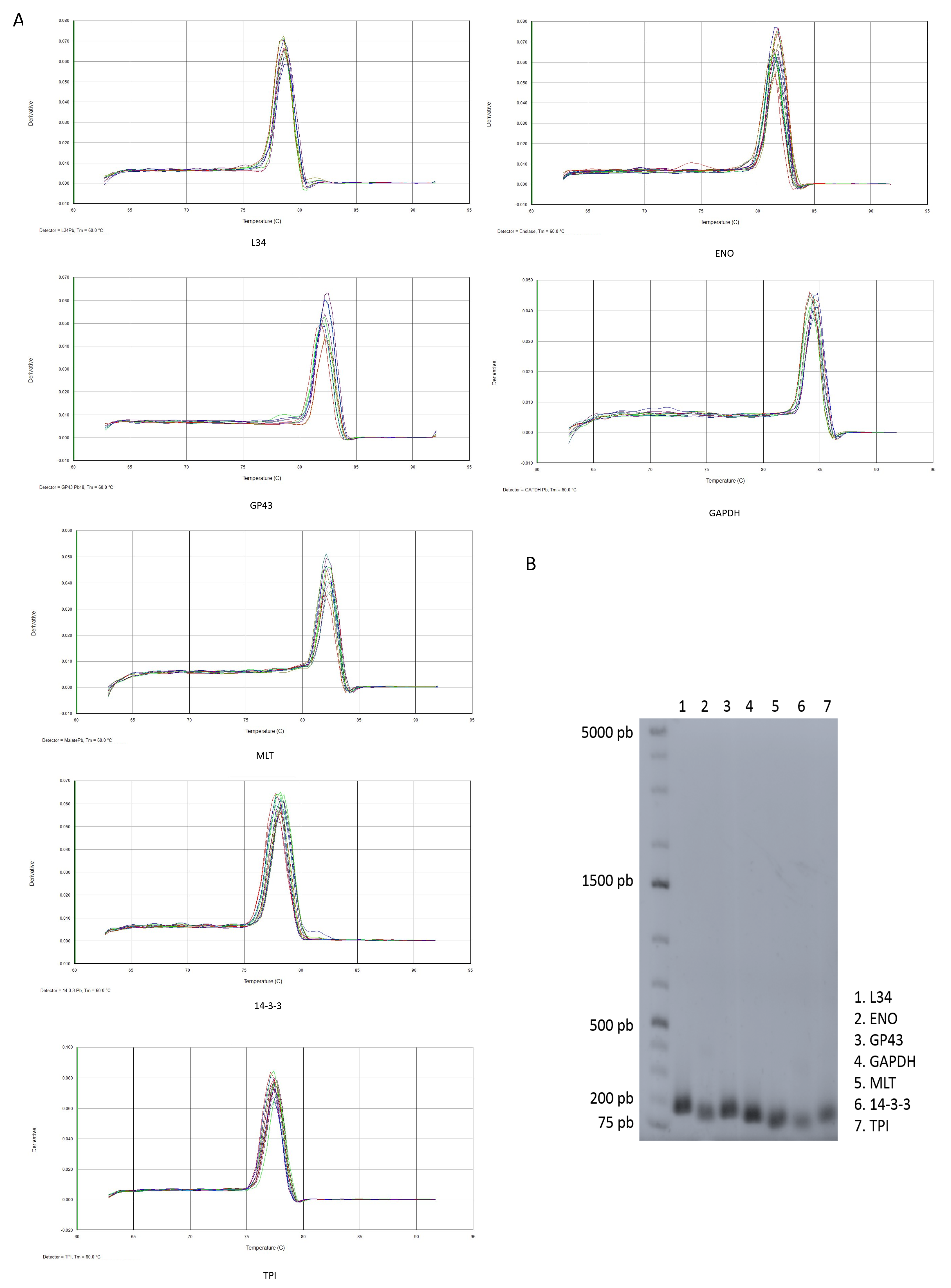

Supplement: Supplementary Figure S1 — Specific amplification of the studied adhesin genes by real-time PCR. (A) Melting curve analysis of all of the primers used showing single peaks. (B) Agarose gel electrophoresis of the PCR products showing single bands. [file Image1.TIF]

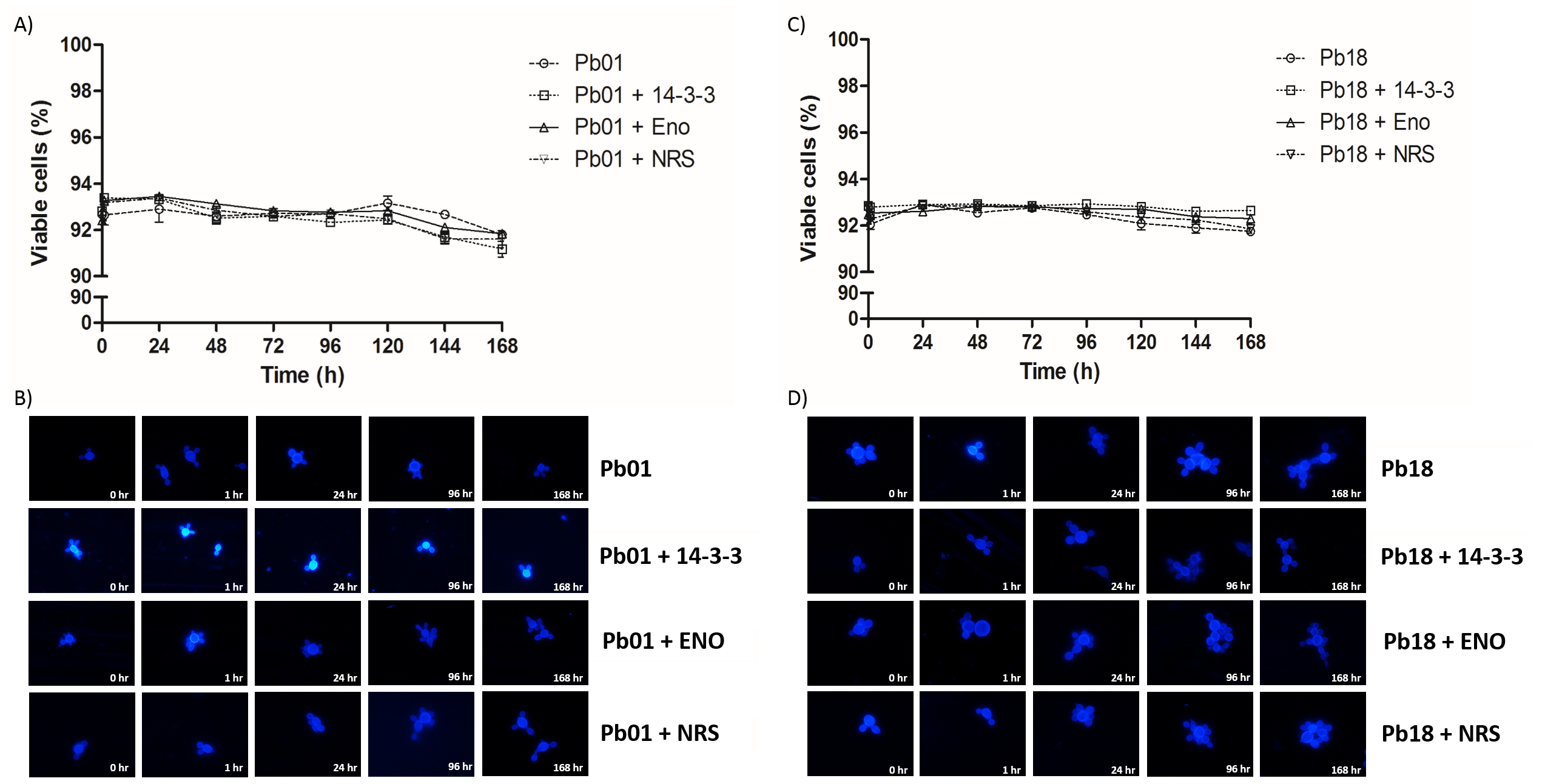

Supplement: Supplementary Figure S2 — Influence of antisera treatment on the survival and morphology of the fungi. Analysis of the influence of fungi treatment with 14-3-3, enolase and NRS antisera. (A) and (C) Viability of P. lutzii and P. brasiliensis, respectively, after treatment with 14-3-3, enolase and NRS antisera. No significant differences were found between the different treatments and the untreated controls. (B) and (D) Analysis of the morphology of P. lutzii and P. brasiliensis, respectively, after treatment with 14-3-3, enolase and NRS antisera (image magnification: 1000x). [file Image2.TIF]
